# Channelized water driven flow of MHD carbon-nanotube nanofluid influenced by rotation, heat source and thermal radiation

**DOI:** 10.1371/journal.pone.0295406

**Published:** 2023-12-27

**Authors:** Moniba Shams, Sofia Sarwar

**Affiliations:** 1 NUST Business School, National University of Sciences and Technology, Islamabad, Pakistan; 2 School of Natural Sciences, National University of Sciences and Technology, Islamabad, Pakistan; United Arab Emirates University, UNITED ARAB EMIRATES

## Abstract

The efficiency enhancements of thermal energy systems are made with advancements made in the effective use of thermal solar collectors, operating fluid and the introduction of curved and transparent solar panels. In this paper, we present a prototype theoretical/mathematical model for the carbon nanotube-based curved solar panels combined with the solar thermal collector and the porous rotating channel. The analysis is carried out to study the effect of transversely applied magnetic, rotation of the porous channel, linear thermal radiation and the uniformly distributed heat source on the heat transfer characteristics of the single-walled (*SW*_*CNT*_) and multi-walled carbon nanotubes (*MW*_*CNT*_). Due to the nonlinearity of the governing momentum and the heat transport equations and the limitation of the exact methods, numerical similarity solutions are obtained for the boundary value problem using the MATLAB function *bvp*4*c*. Influences of different parameters are observed through graphs on the nanofluid flow and temperature profiles. The velocity profile exhibits dual behavior for rising the nanoparticles’ volume fraction, the magnetic parameter, rotation, and the Reynolds number. The temperature profile increases with increasing nanoparticles and heat source parameters and decreases for increasing suction, rotation, Reynolds number, and thermal radiation. In some cases, flow profiles for *SW*_*CNT*_ exceed those of *MW*_*CNT*_.

## Introduction

An increase in industrialization, automation, requirements for heating and cooling, and transportation has increased the global electricity demand. Electricity produced by fossil fuels is the major source of production and fossil fuel reserves are depleting at a rapid pace. Moreover, there are serious environmental concerns associated with the electricity generated through fossil fuels [1, 2]. The increased living requirements for heating and cooling of homes, commercial and residential buildings, businesses and industrial units made HVAC (heating, ventilation, and air conditioning) systems the major consumer of electricity. In developed countries more than 50% of electricity is used by the HAVC structures [3–6]. The introduction of electricity-green HVAC structures that do not rely upon fossil fuels is a key to decreasing electricity intake. Moreover, there is an urgent need to explore and develop new sources for electricity production through sustainable/renewable sources. The generation of electricity through the sun’s light and energy is the greenest, most sustainable, most abundant, and cheapest source available on Earth. The potential electricity that can be generated through solar light or energy is more than 200 times the global need [7–9]. There are two major types of solar electricity production systems are in use, photovoltaic solar panels, and solar thermal collectors. Solar photovoltaic (or PV) generation converts daylight into semiconductors, whereas solar thermal generates energy through the use of the sun’s heat. The introduction of solar HVAC systems based on PV solar panels is considered a breakthrough. The HVAC system consists of solar panels to generate direct current (DC), and the DC air-conditioners, are used as cooling and heating device. In the present work, a prototype mathematical model is proposed to include nanofluid-based solar thermal collectors with rotating channels to the curved and/or transparent PV solar panels in solar HVAC system. The analysis is carried out to study the effect of an introduction of rotating flat surfaces within the solar thermal reservoir filled with carbon-nanotubes-based nanofluid. The heat transfer characteristics are studied including transversely applied magnetic, linear thermal radiation and the uniformly distributed heat source.

Carbon nanotubes (CNT) are cylindrical-shaped nanoparticles suspended in a base fluid like water or kerosene oil to create CNT-nanofluid. The idea of CNT was first proposed by Iijima [10]. Since then extensive research is conducted to study the composition of CNTs, their structural strength, enhanced physical, thermal, and conducting properties, tensile strength, and stability, and applications in different industrial setups. Major applications of CNT include air and water filtration, nanoscale molecular electronics, solar thermal systems etc. A review of the development of CNT-nanofluids can be found in the paper of Kumanek and Janas [11]. Zhang et al. [12] and Xie et al. [13, 14] elaborated on the thermal properties and stability of the carbon nanotubes. More recently, Shafiq et al. [15] and Ramesh [16] established a detailed analysis of single-walled (*SW*_*CNT*_) and multi-walled (*MW*_*CNT*_) carbon nanotubes in the presence of magnetohydrodynamic nanofluid flow. These two types of carbon nanotubes are differentiated in their wall structure and due to this, the nanofluid bears different thermal properties. In particular, *SW*_*CNT*_ are used widely in thermal solar panels due to their high absorption capacity and high-temperature resistance. Nowadays, the thin film of nanofluid is used in the construction of solar panels to increase their efficiency so that a large amount of light is absorbed for a broader range of the solar spectrum. Discussions on various *SW*_*CNT*_ and *MW*_*CNT*_ related research problems are in the references [17–26]. In the case of thermal solar collectors, volume, shape, size and structure of nanoparticles, operating base fluid, externally applied forces, magneto-hydrodynamics (MHD), heat source, heat radiation, geometry, and the flow pattern play their part to improve or determine the overall efficiency of the system. It is important to study the combined effect of these parameters. In [27, 28] the authors showed the presence of a magnetic field enhances the thermal conductivity and heat transfer in the nanofluid. Chamkha and Aly [29] extended the work of previous researchers and describe the effect of heat generation and absorption on the MHD flow of the nanofluid. Haq et al. [30, 31] investigated the effect of the volume fraction of carbon nanotubes on heat transfer over a stretching sheet. Motsumi and Makinde [32], Shehzad [33] and Ramesh et al. [34–36] studied the effect of thermal radiation on the nanofluid. It was observed that both the velocity and temperature of the nanofluid increase with the increase in radiation parameters. Moreover, the radiation generally increases the temperature of the fluid which, in turn, increases the thermal conductivity of the fluid. However, this also depends on the net heat transfer rate of the fluid. The effect of thermal radiation on the MHD flow of the nanofluid is also reported by many researchers, for example, [37–40]. Hussain et al. [41] investigated the rotation effect on the fluid flow of carbon nanotubes. It was observed that in comparison with *MW*_*CNT*_, *SW*_*CNT*_ have reduced drag and a high rate of heat transfer. In a recent study, Jawed et. al [42] described the effect of radiation on the rotating flow of carbon nanotubes. Literature such as [43–60] involves the study of the flow of nanotubes in a rotating frame of reference.

The ongoing trend of miniaturizing electronic devices, those involving rotational components and increasing their power densities leads to a significant generation of heat during their operation. Excessive heat accumulation can have adverse effects on their performance reliability and can even result in premature failure. It is crucial to have efficient cooling mechanisms to dissipate the generated heat and maintain the devices’ temperature within safe operating limits. Recently, scientists and researchers have been exploring the utilization of nanofluids. within cooling systems as a potential solution to enhance heat transfer efficiency. In the present research a prototype mathematical model is presented, incorporating a fluid-filled with a uniform concentration of multi-wall/single-walled carbon nanotubes, along with the introduction of moving parts and externally applied forces, thermal radiation, and heat generation/absorption effects. The authors firmly believe that these additions have the potential to enhance the system’s efficiency significantly and have not been reported in the literature before. The primary objective of this research is to qualitatively comprehend the impact of various parameters on the thermal performance of the system. Please see the references [21, 23, 61, 62]. The major features of this study are listed as under:

- Rotating channel filled with the uniform concentration of multi-walled/single-walled carbon nanotubes.
- Incorporation of applied transfer magnetic field, linear thermal radiation and the heat source on the rate of heat transfer and the velocity gradient at the boundary and within the flow regime of the rotating plates.
- Stretching on the lower place and the suction/injection in the medium.

The MATLAB package *bvp*4*c* is used to find numerical solutions to the boundary value problem. Nanoparticle volume fraction (*ϕ*), injection/suction parameter (*S*), Reynolds number *A*_1_, rotation parameter *A*_2_, magnetic parameter (*M*), rate of heat generation or absorption coefficient (*Q*) and thermal radiation (*Nr*) are assumed to influence the fluid flow, fluid temperature, the local Nusselt number and the skin friction coefficient. These effects are discussed and analyzed in detail. Graphical results depict fluid velocity components, and temperature, whereas tabular results are presented for the heat transfer rate, and drag for various representative values of governing parameters.

## Materials and methods

**Fig 1:**
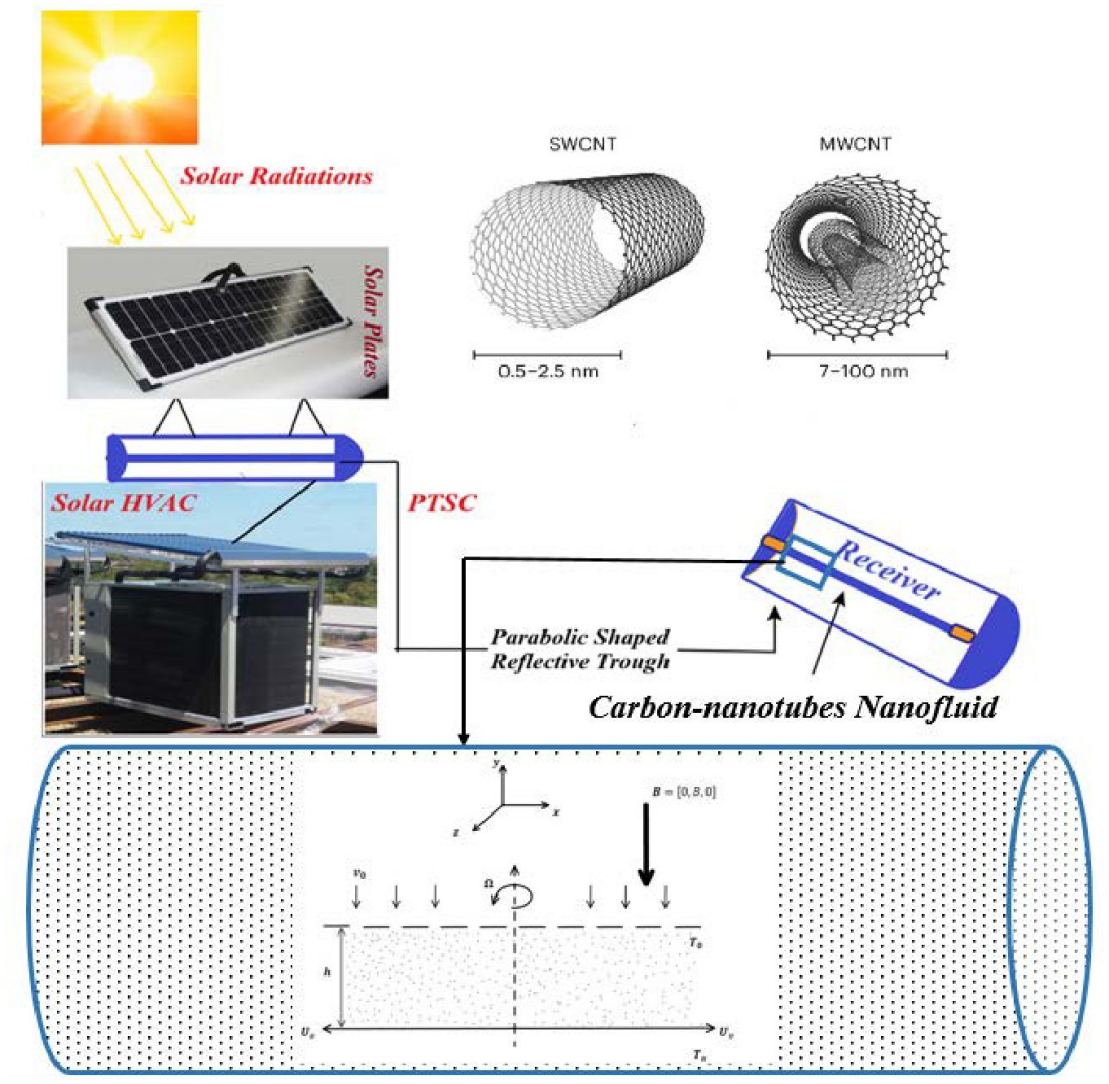
Schematic figure of the problem.

The simplified mathematical model considered here involves a laminar, steady, and incompressible flow of an MHD carbon-nanotubes-based nanofluid flow between a rotating horizontal channel. The channel comprises infinite plates with the *x*-axis along the direction of flow and the *y*-axis is perpendicular to it. Plates are separated by the distance *h* and are rotating with the constant angular velocity Ω. The lower plate is also stretched by a velocity of the form $U_{v}=mx\left(m>0\right)$ in the horizontal direction and the upper plate is porous with fluid attaining constant suction/injection velocity *v*_0_. Here *m* is the constant of proportionality. A uniform constant magnetic field *B*_0_ is applied parallel to the *y*-axis and the induced magnetic field is considered negligible in comparison to the applied magnetic field. The temperature at the upper plate is *T*_0_ and the lower surface attains the temperature *T*_*a*_. The temperature of the lower plate is greater than the upper, i.e *T*_*a*_ > *T*_0_. Finally, the mathematical model is considered in the presence of uniform, linearly distributive radiative heat flux *q*_*r*_ and the constant heat source *Q*_0_. Fig 1 gives the simplified schematic diagram of the problem and Table 1 lists the thermophysical characteristics of the base fluid and the single-walled and multi-walled carbon nanotubes.

**Table 1:** Thermophysical properties of the base fluid and CNTs [50].

Physical properties	Base fluid (water)	*MW*_*CNT*_	*SW*_*CNT*_
*Pr*	6.2		
*κ* (*W*/*mK*)	0.613	3000	6600
*ρ* (*kg*/*m*^3^)	997	1600	2600
*Cp* (*J*/*kgK*)	4179	796	425

In the light of above assumptions the momentum equation is [41, 51]

$$\begin{array}{c} \rho_{nf}(\frac{\;dU}{\;dt}+2\Omega \times U+\Omega \times \left(\Omega \times r\right))=\text{div}\;T, \end{array}$$ (1)

where $U=[U_{1}\left(x,y\right),U_{2}\left(x,y\right),U_{3}\left(x,y\right)],$ is the steady state velocity vector, **T** is the Cauchy stress tensor, **Ω** = [0, Ω, 0], is the angular velocity and *ρ*_*nf*_ is the effective density of the nanofluid. The terms 2(**Ω** × **U**) and (**Ω** × **Ω** × **r**) represent the Coriolis and centripetal forces, respectively. Both the Coriolis and centripetal forces are perpendicular to **U** and **Ω**. The centripetal force is directed towards the y-axis (axis of rotation). In component form, the momentum equation can be written as

$$\begin{array}{cc} & \frac{\partial U_{1}}{\partial x}+\frac{\partial U_{2}}{\partial y}=0, \end{array}$$ (2)

$$\begin{array}{cc} & \rho_{nf}\left(U_{1}\frac{\partial U_{1}}{\partial x}+U_{2}\frac{\partial U_{1}}{\partial y}+2\Omega U_{3}\right)=-\frac{\partial p^{*}}{\partial x}+\mu_{nf}\frac{\partial^{2}U_{1}}{\partial y^{2}}-\sigma_{nf}B_{0}^{2}U_{1}, \end{array}$$ (3)

$$\begin{array}{cc} & \rho_{nf}\left(U_{1}\frac{\partial U_{2}}{\partial x}+U_{2}\frac{\partial U_{2}}{\partial y}\right)=-\frac{\partial p^{*}}{\partial y}+\mu_{nf}\frac{\partial^{2}U_{2}}{\partial y^{2}}, \end{array}$$ (4)

$$\begin{array}{cc} & \rho_{nf}\left(U_{1}\frac{\partial U_{3}}{\partial x}+U_{2}\frac{\partial U_{3}}{\partial y}-2\Omega U_{1}\right)=\mu_{nf}\frac{\partial^{2}U_{3}}{\partial y^{2}}-\sigma_{nf}B_{0}^{2}U_{3}. \end{array}$$ (5)

The subscripts *nf* represents the nanofluid. In the above, $p^{*}=p-\left(\frac{\Omega x^{2}}{2}\right)$, *μ*_*nf*_ is the dynamic viscosity, and the electrical conductivity of the nanofluid respectively. Following [52] the thermal energy equation can be written as

$$\begin{array}{c} {\left(\rho C\right)}_{nf}\left(U_{1}\frac{\partial T}{\partial x}+U_{2}\frac{\partial T}{\partial y}\right)=k_{nf}\left(\frac{\partial^{2}T}{\partial x^{2}}+\frac{\partial^{2}T}{\partial y^{2}}\right)-\frac{\partial q_{r}}{\partial y}+Q_{0}\left(T-T_{a}\right), \end{array}$$ (6)

where (*ρC*)_*nf*_ is the heat capacity of the nanofluid, $q_{r}=-\frac{4\sigma^{*}}{3k^{*}}\frac{\partial T^{4}}{\partial y}$ is the Rosseland’s radiative heat flux [53], σ* is Stefan–Boltzman constant, k* is the mean absorption coefficient and *Q*_0_ is the heat source parameter. The density, dynamic viscosity, heat capacity, thermal conductivity and electrical conductivity of the nanofluid are [41, 45]

$$\begin{array}{c} \rho_{nf}=\left(1-\phi \right)\rho +\phi \rho_{cnt},\;\mu_{nf}=\frac{\mu }{{\left(1-\phi \right)}^{2.5}},\;{\left(\rho C\right)}_{nf}=\left(1-\phi \right)\left(\rho C\right)+\phi {\left(\rho C\right)}_{cnt}, \end{array}$$ (7)

$$\begin{array}{c} \frac{\kappa_{nf}}{\kappa }=\frac{1-\phi +2\phi (\frac{\kappa_{cnt}}{\kappa_{cnt}-\kappa })\;\text{ln}\;(\frac{\kappa_{cnt}+\kappa }{2\kappa })}{1-\phi +2\phi (\frac{\kappa_{cnt}}{\kappa_{cnt}-\kappa })\;\text{ln}\;(\frac{\kappa_{cnt}+\kappa }{2\kappa })},\;\frac{\sigma_{nf}}{\sigma }=1+\frac{3(\frac{\sigma_{cnt}}{\sigma }-1)\phi }{(\frac{\sigma_{cnt}}{\sigma }+2)-(\frac{\sigma_{cnt}}{\sigma }-1)\phi }. \end{array}$$ (8)

Here, the subscript *cnt* specifies the terms relevant to the carbon nanotubes. *ρ* is the density, *μ* is the dynamic viscosity, (*ρC*) the specific heat capacity, *κ* is the thermal conductivity and *σ* the electrical conductivity of the base fluid. The initial and boundary conditions assumed for the given problem are

$$\begin{array}{cc} & U_{1}=U_{v}=mx,\;U_{2}=U_{3}=0,\;T=T_{a},\;at\;y=0, \end{array}$$ (9)

$$\begin{array}{cc} & U_{1}=0,\;U_{2}=-v_{0},\;U_{3}=0,\;-\kappa \frac{\partial T}{\partial y}=h\left[T-T_{a}\right],\;at\;y=h, \end{array}$$ (10)

where *m* and *v*_0_ represents the convective heat transfer coefficient and the uniform suction/injection velocity, respectively.

The solution to governing boundary value problem (2)–(10) is difficult due to the non-linearity in the problem and the limitation of existing analytical methods. Therefore the similarity type numerical solution is sought in this work. Following [41, 52], similarity solutions are assumed to satisfy the equations

$$\begin{array}{c} U_{1}=mxf^{\prime }\left(\eta \right),\;U_{2}=-mhf\left(\eta \right),\;U_{3}=mxg\left(\eta \right),\;\theta \left(\eta \right)=\frac{T-T_{0}}{T_{a}-T_{0}},\;\eta =\frac{y}{h}, \end{array}$$ (11)

where *η* is the dimensionless similarity variable and prime denotes the differentiation of the function with respect to *η*. By eliminating the pressure gradient in Eqs (3) and (A.3) in S1 Appendix and using Eq (11), the system of partial differential Eqs (2)–(6) is transformed to the following system of ordinary differential equations. That is,

$$\begin{array}{cc} & f^{iv}-\varepsilon_{1}\zeta A_{1}\left(f^{\prime }f^{\prime \prime }-ff^{\prime \prime \prime }\right)-2A_{2}\varepsilon_{1}\zeta g^{\prime }-A_{3}\zeta Mf^{\prime \prime }=0, \end{array}$$ (12)

$$\begin{array}{cc} & g^{\prime \prime }-\varepsilon_{1}A_{1}\left(gf^{\prime }-fg^{\prime }\right)+2\varepsilon_{1}A_{2}f^{\prime }-A_{3}Mg=0, \end{array}$$ (13)

$$\begin{array}{cc} & \theta^{\prime \prime }(1+\frac{Nr}{\varepsilon_{3}})+\frac{A_{1}}{\varepsilon_{3}}\varepsilon_{2}Prf\left(\eta \right)\theta^{\prime }+\frac{Q}{\varepsilon_{3}}\theta =0. \end{array}$$ (14)

For details, see S1 Appendix. Various dimensionless parameters appearing in Eqs (12–14) are

$$\begin{array}{cc} & A_{1}=\frac{mh^{2}}{\nu },\;A_{2}=\frac{\Omega h^{2}}{\nu },\;A_{3}=\frac{\sigma_{nf}}{\sigma },\;\zeta ={\left(1-\phi \right)}^{2.5}\\ & Q=\frac{Q_{0}h^{2}}{k},\;M=\frac{\sigma h^{2}B_{0}^{2}}{\rho \nu }{\left(1-\phi \right)}^{2.5},\;Nr=\frac{16\sigma }{3k^{*}}\frac{T_{a}^{3}}{k},\\ & \varepsilon_{1}=\left[\left(1-\phi \right)+\phi \frac{\rho_{cnt}}{\rho }\right]\zeta ,\;\varepsilon_{2}=\left[\left(1-\phi \right)+\phi \frac{{\left(\rho C\right)}_{cnt}}{\rho C}\right],\\ & \varepsilon_{3}=\frac{\kappa_{nf}}{\kappa }=\frac{1-\phi +2\phi \left(\frac{\kappa_{cnt}}{\kappa_{cnt}-\kappa }\right)\;\text{ln}\;\left(\frac{\kappa_{cnt}+\kappa }{2\kappa }\right)}{1-\phi +2\phi \left(\frac{\kappa }{\kappa_{cnt}-\kappa }\right)\;\text{ln}\;\left(\frac{\kappa_{cnt}+\kappa }{2\kappa }\right)}, \end{array}$$ (15)

where *ν* is the kinematic viscosity of the base fluid and *σ* is the electrical conductivity of the nanofluid. Boundary conditions (10) after introduction of similarity variables (11) transformed into

$$\begin{array}{c} f\left(0\right)=0,\;f^{\prime }\left(0\right)=1,\;g\left(0\right)=0,\;\theta \left(0\right)=1, \end{array}$$ (16)

at *η* = 0 (corresponding to *y* = 0) and

$$\begin{array}{c} f\left(1\right)=S,\;f^{\prime }\left(1\right)=0,\;g\left(1\right)=0,\;\theta^{\prime }\left(1\right)=\frac{-Bi(\theta -1)}{\varepsilon_{3}}, \end{array}$$ (17)

at *η* = 1 (corresponding to *y* = *h*). Here, *S* is the dimensionless suction/injection parameter and $Bi=\frac{ha}{\kappa }$ is the Biot number. Physical parameters of interest are the skin friction coefficient *C*_*f*_ and the local Nusselt number *Nu*. These are defined as

$$\begin{array}{c} C_{f}=\frac{\tau_{s}}{\rho_{nf}U_{v}^{2}},\;Nu=\frac{xq_{s}}{\kappa_{nf}\left(T_{a}-T_{0}\right)}. \end{array}$$ (18)

In the above *τ*_*s*_ is the shear stress and *q*_*s*_ is the heat flux. Following [54]

$$\begin{array}{c} \tau_{s}=\mu_{nf}\frac{\partial U_{1}}{\partial y},\;q_{s}=-\kappa_{nf}\frac{\partial T}{\partial y}+\frac{16\sigma^{*}T_{a}^{3}}{3k^{*}}\frac{\partial T}{\partial y}. \end{array}$$ (19)

Using expressions from Eq (11) the shear stress and the heat flux from Eq (18) transform to

$$\begin{array}{cc} & C_{f}^{*}=\frac{U_{v}h}{\nu_{f}}C_{f}=\frac{f^{\prime \prime }\left(\eta \right)}{(\frac{1}{\left(1-\phi \right)+\phi \frac{\rho }{\rho_{nf}}}){\left(1-\phi \right)}^{2.5}}, \end{array}$$ (20)

$$Nu_{x}^{*}=\frac{h}{x}Nu_{x}=-\theta^{\prime }(\eta )(1+\frac{Nr}{\varepsilon_{3}}),$$ (21)

and both are calculated at *η* = 0 (*y* = 0) and *η* = 1 (*y* = *h*).

The two-point boundary value problem, as given in Eqs (12–14) along with boundary conditions (16 and 17) is difficult to solve analytically. Therefore numerical solutions are sought for the modeled problem using the MATLAB function *bvp*4*c*. Details of Matlab *bvp*4*csolver* are extensively available in the literature and is not the main focus of the present research. The authors take the liberty and not discuss these details here. To verify the accuracy of results obtained by the present *bvp*4*c* numerical scheme, a comparison of numerical results is presented in Table 2 for the skin friction coefficient and the local Nusselt number with those presented in [41]. The results are found to be in good agreement and validate the accuracy of present scheme.

**Table 2:** Comparison of the skin friction coefficient $C_{f}^{*}$ and local Nusselt number $Nu_{x}^{*}$ at *η* = 0 and *η* = 1 for different values of *S* with results by Hussain et al. [41], when *A*_1_ = 0.5, *A*_2_ = 1, *ϕ* = 0.2, *Pr* = 6.2 and rest of the parameters vanish.

S	Hussain et al. [41]	Present work	Hussain et al. [41]	Present work
	$C_{f}^{*}$ at *η* = 0		$C_{f}^{*}$ at *η* = 1	
−1	−12.99946	−12.9989	10.99095	10.9905
0	−5.33832	−5.33811	2.61972	2.61962
1	2.77224	2.77213	−5.19788	−5.19767
	$Nu_{x}^{*}$ at *η* = 0.		$Nu_{x}^{*}$ at *η* = 1.	
−1	0.95029	0.950284	1.16778	1.1678
0	1.02506	1.02506	0.98349	0.983484
1	1.10111	1.10111	0.82224	0.822262

## Results

**Fig 2:**
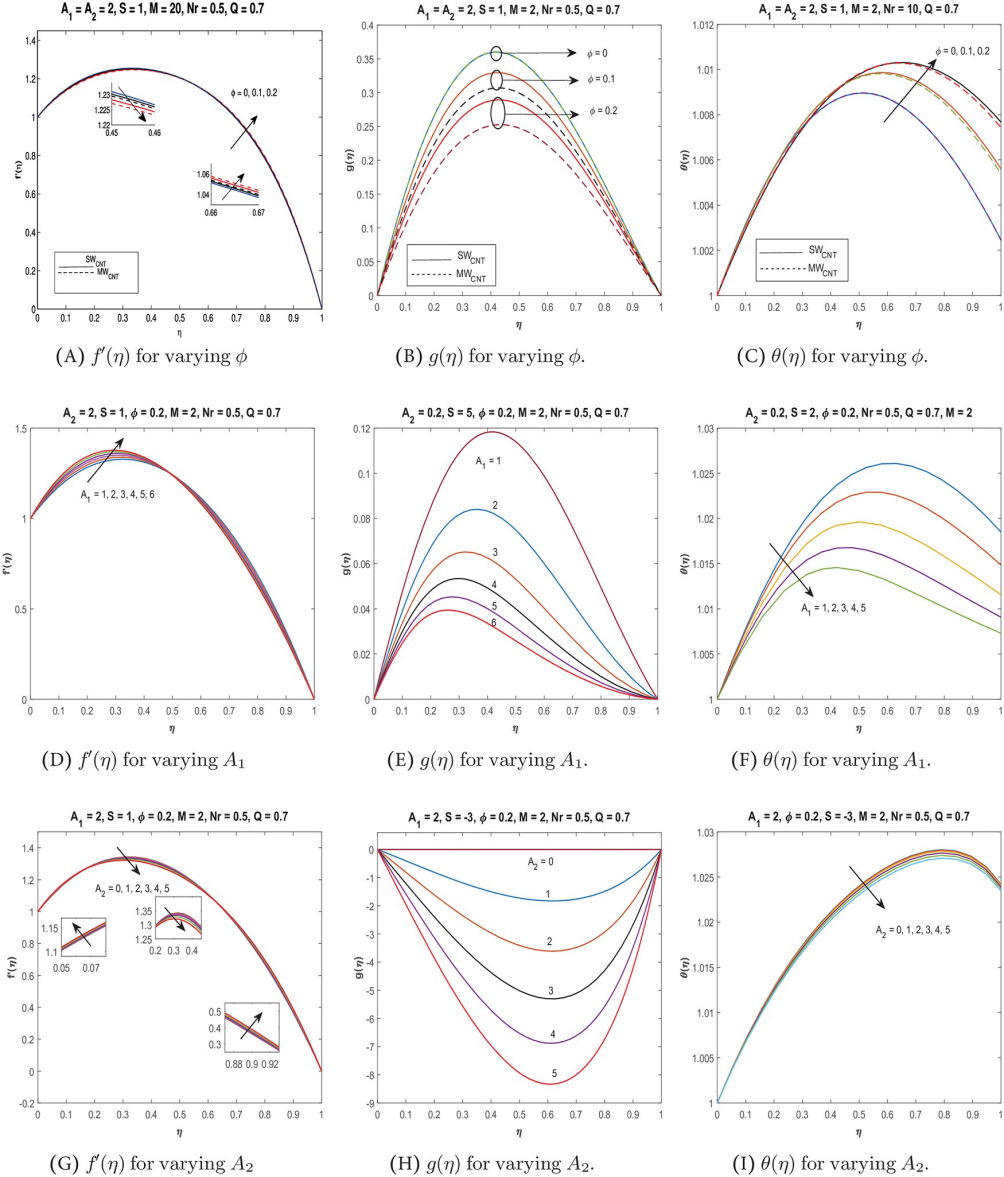
Variation of *f*′(*η*), *g*(*η*) and *θ*(*η*) due to parameters *ϕ*, *A*_1_ and *A*_2_.

**Fig 3:**
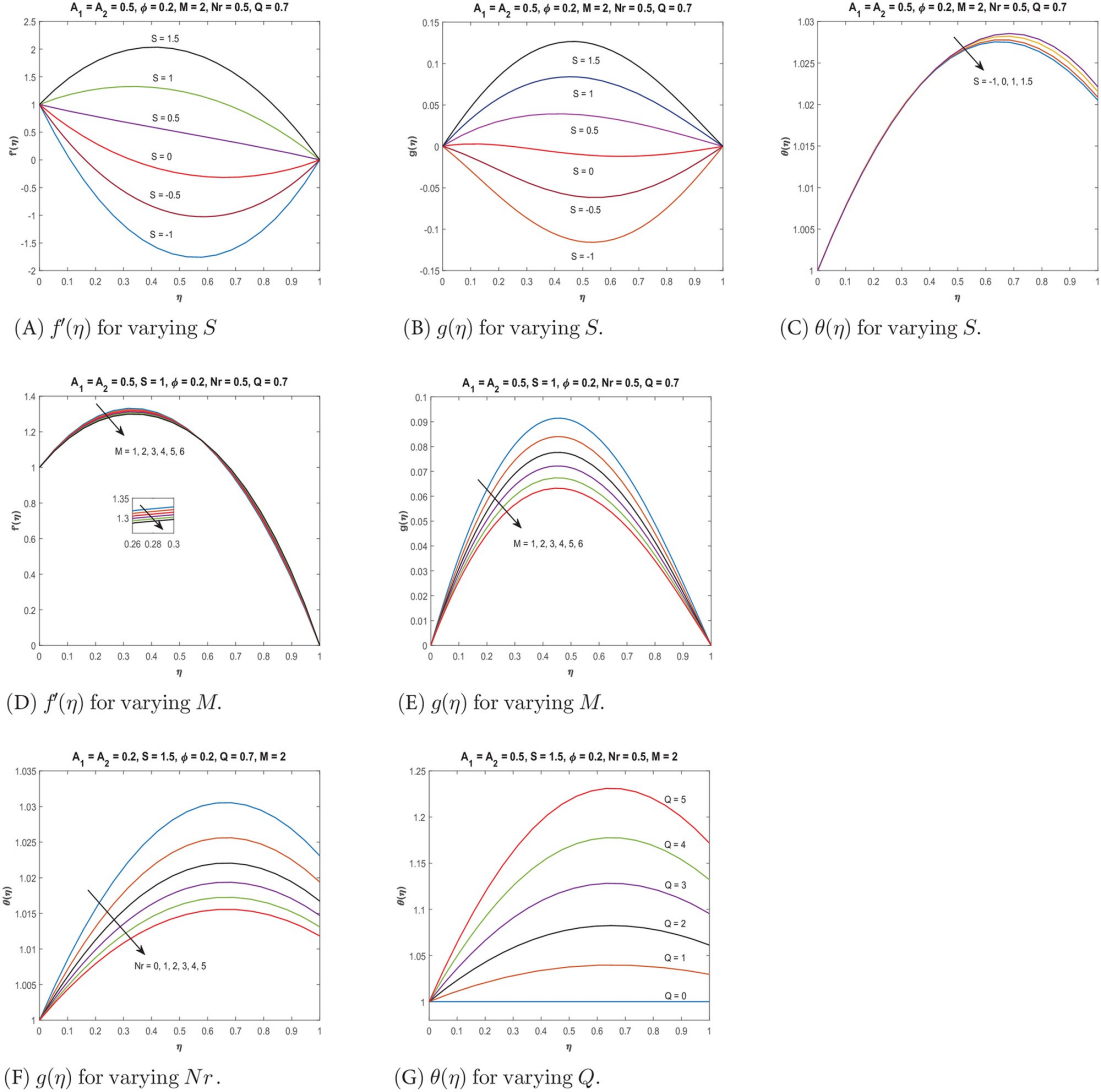
Variation of *f*′(*η*), *g*(*η*) and *θ*(*η*) due to parameters *S*, *M*, *Nr* and *Q*.

Numerical results obtained using *bvp*4*c* numerical scheme are presented graphically in Figs 2 and 3 for the water-based nanofluid including both single-wall carbon nanotubes *SW*_*CNT*_ and multi-wall carbon nanotubes *MW*_*CNT*_. Readers may refer to S1 File for details on the software used for graphing. Computations are carried out for velocity components *f*′(*η*) and *g*(*η*), temperature profile *θ*(*η*), skin friction coefficient $C_{f}^{*}$ and the local Nusselt number $Nu_{x}^{*}$ with variation in important governing parameters like nanoparticles volume fraction *ϕ*, suction/injection parameter *S*, Reynolds number *A*_1_, rotation parameter *A*_2_, the magnetic parameter *M*, thermal radiation parameter *Nr* and heat generation or absorption parameter *Q*. Numerical computations carried out for the skin friction coefficient and the local Nusselt number at *η* = 0 and *η* = 1, are presented in Tables 3 and 4. In particular, for fixed *S*, Table 3 gives the data for the skin friction coefficient $C_{f}^{*}$ for variation in *ϕ*, *M*, *A*_1_ and *A*_2_. Table 4 present data for the local Nusselt number $Nu_{x}^{*}$. The calculations are made for injection (*S* < 0), no suction/injection (*S* = 0), and suction (*S* > 0).

**Table 3:** Results of skin friction coefficient for *SW*_*CNT*_ and varying choices of *ϕ*, *S*, *M*, *A*_1_ and *A*_2_ with *Bi* = 10 and *Pr* = 6.2 at *η* = 0 and *η* = 1.

*S*	*ϕ*	*M*	*A*_1_	*A*_2_	Skin friction coefficient at *η* = 0	Skin friction coefficient at *η* = 1
-1	0.0	2	0.5	0.5	-10.2194	8.51706
	0.1				-11.4843	9.49932
	0.2				-13.582	11.129
-1	0.2	1	0.5	0.5	-13.2767	11.0465
		2			-13.582	11.129
		3			-13.8812	11.2126
-1	0.2	2	0.5	0.5	-13.582	11.129
			1		-13.332	11.5239
			2		-12.8302	12.3941
-1	0.2	1	0.5	0.5	-13.582	11.129
				1	-13.6094	11.1491
				2	-13.719	11.2295
0	0.0	2	0.5	0.5	-4.30362	1.91557
	0.1				-4.81925	2.15016
	0.2				-5.67422	2.53909
0	0.2	1	0.5	0.5	-5.50537	2.5783
		2			-5.67422	2.53909
		3			-5.83904	2.50212
0	0.2	2	0.5	0.5	-5.67422	2.53909
			1		-5.71563	2.51784
			2		-5.79824	2.47611
0	0.2	1	0.5	0.5	-5.67422	2.53909
				1	-5.67922	2.53868
				2	-5.69914	2.53707
1	0.0	2	0.5	0.5	2.04464	-4.14171
	0.1				2.27874	-4.65604
	0.2				2.66702	-5.50936
1	0.2	1	0.5	0.5	2.71019	-5.34374
		2			2.66702	-5.50936
		3			2.62633	-5.6715
1	0.2	1	0.5	0.5	2.71019	-5.34374
			1		2.81369	-5.22796
			2		3.01939	-5.02205
1	0.2	1	0.5	0.5	2.71019	-5.34374
				1	2.72464	-5.36509
				2	2.7824	-5.45031

**Table 4:** Results of local Nusselt number for for *SW*_*CNT*_ and different values of *ϕ*, *S*, *Nr*, *Q*, *A*_1_ and *A*_2_ with *Bi* = 10 and *Pr* = 6.2 at *η* = 0 and *η* = 1.

*S*	*ϕ*	*Nr*	*Q*	*A*_1_	*A*_2_	local Nusselt number at *η* = 0	local Nusselt number at *η* = 1
-1	0.0	0.5	0.5	0.5	0.5	0.301895	0.319987
	0.1					0.109816	0.074568
	0.2					0.0649132	0.032436
-1	0.2	0.5	0.5	0.5	0.5	0.0649132	0.032436
		1				0.0648088	0.0323131
		2				0.0646422	0.0321174
-1	0.2	0.5	0.5	0.5	0.5	0.0649132	0.032436
			1			0.131662	0.0660433
			2			0.27106	0.137031
-1	0.2	0.5	0.5	0.5	0.5	0.0649132	0.032436
				1		0.0653095	0.0333701
				2		0.0659092	0.0349731
-1	0.2	0.5	0.5	0.5	0.5	0.0649132	0.032436
					1	0.0649119	0.0324356
					2	0.0649072	0.0324344
0	0.0	0.5	0.5	0.5	0.5	0.293849	0.237746
	0.1					0.109518	0.0685951
	0.2					0.0652628	0.0315805
0	0.2	0.5	0.5	0.5	0.5	0.0652628	0.0315805
		1				0.0651253	0.0315236
		2				0.064908	0.0314334
0	0.2	0.5	0.5	0.5	0.5	0.0652628	0.0315805
			1			0.13237	0.0642906
			2			0.27251	0.133347
0	0.2	0.5	0.5	0.5	0.5	0.0652628	0.0315805
				1		0.06609	0.031743
				2		0.0677575	0.0320633
0	0.2	0.5	0.5	0.5	0.5	0.0652628	0.0315805
					1	0.0652624	0.0315804
					2	0.0652609	0.0315801
1	0.0	0.5	0.5	0.5	0.5	0.277623	0.167682
	0.1					0.108426	0.0621792
	0.2					0.0654577	0.0305971
1	0.2	0.5	0.5	0.5	0.5	0.0654669	0.0305971
		1				0.0653226	0.0306265
		2				0.0650909	0.0306707
1	0.2	0.5	0.5	0.5	0.5	0.0654577	0.0305971
			1			0.132754	0.0622718
			2			0.27325	0.129088
1	0.2	0.5	0.5	0.5	0.5	0.0654577	0.0305971
				1		0.0662431	0.0296135
				2		0.0670672	0.0272519
1	0.2	0.5	0.5	0.5	0.5	0.0654577	0.0305971
					1	0.0654579	0.0305971
					2	0.0654586	0.0305974

## Discussion

This section presents a discussion of velocity and temperature profiles, taking into account various effective and governing parameters. The physical reasoning underlying their behavior is explained, with the primary objective of addressing the research questions raised in the section *Introduction*. The section concludes with an analysis of heat transfer rate and velocity gradient at the boundary.

### Nanoparticles concentration *ϕ*

Overall, the effect of increasing the concentration of nanotubes on fluid motion depends on various factors, such as the properties of the nanotubes (size, aspect ratio, surface chemistry, etc.), the properties of the fluid (viscosity, ionic strength, pH, etc.), and the flow conditions (velocity, pressure, geometry, etc.). In some cases, increasing the nanotube concentration can enhance fluid motion, while in other cases, it can lead to increased viscosity and hinder flow. In Fig 2(A)–2(C), the authors investigate the effect of varying the volume fraction of nanoparticles (*ϕ*) in a base fluid on the behaviour of the nanofluid. The results are presented for both single-walled carbon nanotubes (*SW*_*CNT*_) and multi-walled carbon nanotubes (*MW*_*CNT*_).

The velocity of nanofluid in Fig 2A follows the assumed boundary conditions, initially increasing and then diminishing at *η* = 1, which confirms the accuracy of the numerical results. The nanofluid velocity *f*′(*η*) decreases as the nanoparticle volume fraction *ϕ* increases for both *SW*_*CNT*_ and *MW*_*CNT*_ nanofluids, as expected due to the increased viscosity and reduced fluid motion caused by the addition of solid particles. However, over time, the rotational effect becomes more dominant and reverses the behaviour of *f*′(*η*), causing the velocity of the nanofluid far from the lower plate to increase with increasing *ϕ*. The velocity profile *f*′(*η*) is slightly lower for *SW*_*CNT*_ nanofluids compared to *MW*_*CNT*_ nanofluids, while the velocity *g*(*η*) decreases as *ϕ* increases for both types of nanofluids.

Fig 2B illustrates that the *g*(*η*) for *SW*_*CNT*_ displays a greater value in comparison to that of *MW*_*CNT*_. A plausible explanation is that the velocity is higher for *SW*_*CNT*_ than *MW*_*CNT*_ with increasing concentration of nanotubes because *SW*_*CNTs*_ have a higher aspect ratio (length-to-diameter ratio) than *MW*_*CNTs*_. As the concentration of nanotubes increases, the interaction between the nanotubes becomes stronger, and the aspect ratio plays a significant role in determining the velocity. Due to their higher aspect ratio, *SW*_*CNTs*_ are more effective at creating a network that facilitates fluid flow, resulting in a higher velocity compared to *MW*_*CNTs*_.

Fig 2C illustrates how changes in the volume fraction of nanoparticles (*ϕ*) impact the temperature of the nanofluid. The results indicate that as *ϕ* increases, so does the temperature of the nanofluid due to an increase in overall thermal conductivity resulting from the introduction of solid particles. However, when *ϕ* is fixed, the temperature profile for *SW*_*CNT*_ is only slightly higher than that of *MW*_*CNT*_. At higher volume fractions, the interaction between nanoparticles has a more significant impact on thermal conductivity, making the difference between *SW*_*CNT*_ and *MW*_*CNT*_-based nanofluids less significant. The qualitative behaviour of nanofluids based on *SW*_*CNT*_ and *MW*_*CNT*_ is similar, as shown in Fig 2(A)–2(C). Therefore, we only present the graphs for the *SW*_*CNT*_ based nanofluid in the graphs to follow.

### Reynolds number *A*_1_

Curves for velocity profiles *f*′(*η*), *g*(*η*), and temperature profile *θ*(*η*) are presented in Fig 2(D)–2(F), illustrating how they are affected by increasing Reynolds number *A*_1_. As shown in Fig 2D, the velocity of nanofluid initially increases (for 0 ≤ *η* ≤ 0.5) with the raising values of *A*_1_. As the Reynolds number increases, there is more turbulence in the channel flow, which increases fluid velocity. However, when a stretching plate is introduced, the stretching effect of the plate takes over the higher inertial forces, which leads to a decrease in fluid velocity. This effect becomes more pronounced as the Reynolds number increases. At a certain point, the stretching effect becomes dominant, and the fluid velocity starts decreasing after the mean position of the rotating channel. The effect of variation in *A*_1_ is more pronounced for the velocity in the *z*-direction. The nanofluid velocity reduces with ascending values of Reynolds number, and the maximum velocity of the nanofluid keeps shifting towards the lower surface. However, the maximum velocity is lowered with increasing *A*_1_. The behaviour of the velocity component *g*(*η*) in Fig 2E can depend on various factors, including the specific values of the other parameters involved in the fluid flow problem, such as the geometry of the channel, fluid properties, and boundary conditions. As such, the decrease in *g*(*η*) with increasing Reynolds number may not necessarily be a constant or universal behaviour, but rather one that is specific to the particular fluid flow situation being studied. Fig 2F shows that increasing *A*_1_ leads to a decrease in the temperature profile (*θ*(*η*)). At low Reynolds numbers, where the lower surface experiences minimal stretching, the temperature is higher due to the dominance of viscous forces over momentum forces. The maximum temperature occurs between the lower plate and the mean position, and it decreases as the Reynolds number increases.

### Rotation parameter *A*_2_

The velocity component *f*′(*η*) is shown in Fig 2G for increasing values of the rotation parameter *A*_2_. The behaviour of the velocity component is complex, with an initial increase in velocity near the lower plate, followed by a decrease in velocity until it begins to increase again near the upper plate. This suggests that the effect of rotation on the *x*-component of velocity is mixed which is likely due to the combined influence of various factors, such as the flow geometry, the magnetic field, and the fluid properties. When rotation is introduced, it can create a swirling motion within the fluid, which can either enhance or hinder the flow in certain regions. In addition, the presence of magnetic fields can also have a significant impact on flow behaviour, especially in highly conductive fluids. Overall, the interaction between these various factors can lead to a complex and non-linear response of the velocity component to changes in the rotation parameter. Fig 2H shows that increasing *A*_2_ results in a decreasing trend in the velocity component *g*(*η*). The rotation effect is weak near the lower and upper walls of the channel, and the nanofluid velocity is lowest in these regions. Regardless of the specific value of *A*_2_, the maximum velocity is observed near the mean position of the plates.

In the absence of rotation (*A*_2_ = 0), the problem reduces to two-dimensional nanofluid flow in a channel with MHD, as can be verified from Eqs (12) and (13). The reason for the observed decrease in temperature profile *θ* with increasing values of the rotation parameter *A*_2_ in Fig 2I is because the introduction of rotation can affect the heat transfer characteristics of the fluid. Specifically, when the fluid is subjected to rotation, it can create a swirling motion that can lead to increased mixing and turbulence, which can in turn enhance the convective heat transfer within the fluid. As a result, the temperature of the fluid may decrease as it flows through the channel, which is consistent with the behaviour observed in Fig 2I. Additionally, the observed opposite behaviour from [41] may be due to differences in the specific setup and parameters used in the two studies.

### Suction and injection parameter *S*

The behaviour of *f*′(*η*) and *g*(*η*) concerning the suction/injection parameter *S* is shown in Fig 3A and 3B. It can be observed that both velocity components increase as the value of *S* increases. This can be explained by the fact that the increased velocity results in a higher shear rate, which reduces the fluid’s viscosity and allows for better flow. Additionally, for higher values of suction (*S* > 0), the nanofluid flow is laminar within the boundary layer, leading to a decrease in friction losses and subsequently a decrease in boundary layer thickness. The variation in velocities is more significant in the middle of the channel, as compared to the region close to the plates, where the effects of rotation and stretching are more prominent. Fig 3C shows the temperature profile of the nanofluid, which decreases with increasing values of the suction parameter *S*. This implies that as the volume flow rate increases, the rate of heat transfer decreases.

### Magnetic parameter *M*

The effect of the magnetic parameter *M* on the velocity profiles *f*′(*η*) and *g*(*η*) is demonstrated in Fig 3D and 3E. The decrease in velocity distribution with increasing magnetic flux is due to the production of a body force called the Lorentz force by the magnetic field. This force opposes the fluid motion, restricting the fluid’s freedom of movement and thereby reducing the velocities. However, this effect is not as prominent in the nanofluid close to the upper plate, and the velocity behavior is reversed.

### Radiation and heat source parameters

Fig 3F and 3G demonstrate the impact of radiation parameter *Nr* and heat source parameter *Q* on the fluid temperature. The results show that an increase in *Nr* leads to a decrease in temperature, whereas an increase in *Q* results in a higher temperature. In the presence of radiation, energy is transferred from the fluid to the surrounding, causing a cooling effect, and leading to a decrease in temperature. On the other hand, as the heat source generates more energy, it increases the temperature of the fluid.

### Nusselt number and skin friction coefficient

This subsection presents an analysis of the effects of various governing parameters on the fluid behavior near the boundary. The magnitude of the skin friction coefficient is determined by the velocity gradient at the boundary layer. A higher velocity gradient leads to a higher skin friction coefficient, while a lower velocity gradient leads to a lower skin friction coefficient. This relationship is due to the fact that a higher velocity gradient signifies a greater rate of velocity change near the surface, resulting in a greater frictional force on the surface. In order to obtain a better understanding of the velocity behavior at the boundary, Table 3 gives the numerical results for skin friction coefficient using Eq (20). Additionally, the convective heat transfer from a surface can be defined by the dimensionless Nusselt number. Thus, the numerical values of the Nusselt number can be found in Table 4, which have been computed using Eq (21). These variations are analyzed briefly for each of the governing parameters below.

*Effect of ϕ*: It is found that for *η* = 0, 1, the skin friction coefficient increases for *S* = −1, 0, 1. Hence, with the increase in the volume fraction of nanoparticles, the resistance to fluid flow increases near the boundaries. This effect travels in the fluid due to a higher concentration of CNT and the flow rate decreases. The rate of heat transfer at the boundary decreases as the volume fraction of nanoparticles increases. The decrease in the velocity profile and the heat transfer rate renders a higher temperature profile of the fluid.

*Effect of S*: For *S* = −1 (injection), varying *ϕ*, $C_{f}^{*}$ decreases at *η* = 0 and increases at *η* = 1, which means less friction at the lower plate and increased friction at the upper plate is experienced. Similar behavior is observed for *S* = 0. For *S* > 0, an increase in *ϕ* results in an increase in $C_{f}^{*}$ at *η* = 0 and hence causes the velocity to decrease near the lower plate, (see Fig 2C). In contrast, $C_{f}^{*}$ decreases at *η* = 1, which causes the velocity to increase near the upper plate. For increasing values of *S*, the local Nusselt number decreases which shows decreasing heat transfer rate and hence a decrease in temperature profile as shown in Fig 3C. *Effect of M*:For *S* = −1, varying *M*, shows decreasing trend in $C_{f}^{*}$ at *η* = 0 and increasing at *η* = 1. Whereas for *S* = 0, $C_{f}^{*}$ decreases at both plates and for *S* = 1, it increases at *η* = 0 and decreases at *η* = 1, which again accounts for the dual behaviour of velocity as captured in Fig 3D. Hence, the velocity profile is different near the two plates in the presence of MHD and the other parameters.

*Effect of A_1_.*: For a given value of *ϕ* and *S* = 1, and increasing the Reynolds number *A*_1_, the behaviour of skin friction coefficient at the lower and upper plates is opposite. The velocity profiles as shown in Fig 2(D) and 2(E), decreases initially owing to the increase in friction with increasing *A*_1_ near the lower plate and increases near the upper plate when friction decreases. The local Nusselt number shows a decreasing behaviour with increasing *A*_1_ and in turn, the temperature profile thus falls (see Fig 2F). *Effect of A_2_*: Linear increase in the rotation parameter *A*_2_ when *S* = 1, increases the skin friction at both upper and lower plates Similarly, the behaviour of the local Nusselt number increases at the two boundaries. This general behaviour is consistent with studies that reported the rotating channel flow. *Effect of* *Q*, *Nr* *and* *Bi*: The heat transfer rate is increasing at both the boundaries with varying heat source parameters *Q* at *S* = 1, and the overall effect of this behavior on the temperature profile can also be seen as increasing in Fig 3G. The heat transfer rate for varying radiation parameter *Nr* at *S* = 1 decreases at *η* = 0 whereas it increases at *η* = 1. The overall effect of this heat transfer is shown in Fig 3F. The temperature profile is found to decrease as *Nr* increases. The heat transfer rate decreases at *η* = 0 for increasing values of *Bi* whereas it is increasing at *η* = 1.

## Conclusions

In this study, a prototype mathematical model is proposed to include nanofluid-based solar thermal collectors with rotating channels to the curved and/or transparent PV solar panels. The analysis is carried out to study the effect of an introduction of rotating flat surfaces within the thermal reservoir filled with carbon-nanotubes-based nanofluid. The heat transfer characteristics are studied including transversely applied magnetic, linear thermal radiation and the uniformly distributed heat source. The solution to the problems is established by using appropriate similarity transformation on the system of partial differential equations. The resulting system of ordinary differential equations is solved using MATLAB with appropriate boundary conditions. The effects of various parameters on velocity and temperature profiles are discussed in detail. Flow drag and rate of heat transfer are observed in tabular form and graphical illustrations are presented. In particular, the study is aimed to answer some pertinent research questions raised in the introduction of the paper. In light of these, the most significant findings are:

- As the volume fraction of nanoparticles; Reynolds number; rotation; magnetic or the suction/injection parameter increase, the velocity shows a dual behavior. This is possible due to changes in the viscosity of the fluid.
- The temperature of the fluid increases with increasing volume fraction of nanoparticles *ϕ* and decreases for suction/injection *S*; Reynolds number *A*_1_ or rotation parameter *A*_2_.
- With suction, as the heat in the system increases, that is with increasing *Q*, the temperature of the fluid rises whereas it drops with increasing radiation parameter *Nr*.
- Nanofluids with *SW*_*CNT*_ show higher velocity profile as compared to those for *MW*_*CNT*_ which indicates that *SW*_*CNT*_ produce less resistance to fluid flow.
- For increasing nanoparticle volume fraction *ϕ*, the velocity gradient and the rate of heat transfer increase.
- For increasing rotation *A*_2_, the skin friction coefficient and Nusselt number increase.
- For increasing magnetic parameter *M*, the skin friction coefficient shows opposite behavior near the upper and the lower plates and hence velocity profile is different near the two plates.

## Future work

The present prototype model presented in this research can be further extended by incorporating the nonlinear aspect of the underlying physical model for the understating of the design engineer. For example, the study of nonlinear thermal radiation and heat source, introduction of hybrid or tri-hybrid nanofluids, viscous dissipation and the Joule’s heating effect, and the pressure driven flow, etc. The introduction of nonlinear parameters will improve the understanding of the efficiency and performance of these systems, and ultimately lead to the development of more advanced and sustainable technologies.

## Supporting information

S1 File MATLAB file for graphs. (PDF)

S1 Appendix (PDF)

## Abbreviations

Ω(*rad*/*s*): Angular Velocity
*α*(*m*^2^/*s*): Thermal Diffusivity
*κ*(*W*/*mK*): Thermal Conductivity
*μ*(*kg*/*ms*): Dynamic Viscosity
*ν*(*m*^2^/*s*): Kinematic Viscosity
*ρ*(*kg*/*m*^3^): Density
*σ**(*W*/*m*^2^*k*^4^): Stefan-Boltzman constant
*A*_1_: Dimensionless Reyolds Number
*A*_2_: Dimensionless Rotation Parameter
*B*_0_(*A*/*m*): Magnetic Field
Bi: Dimensionless Biot Number
*C*_*f*_: Dimensionless Skin Friction Coefficient
*Cp*(*J*/*kgK*): Specific Heat
*k**: Dimensionless Mean Absorption Parameter
M: Dimensionless Magnetic Parameter
Nr: Dimensionless Thermal Radiation Parameter
Nu: Dimensionless Nusselt Number
Pr: Dimensionless Prandtl Number
Q: Dimensionless Heat Source Parameter
*Q*_0_(*J*/*m*^3^*sK*): Heat Source Parameter
*q*_*v*_(*W*/*m*^2^): Heat Flux
*T*_*a*_(*K*): Temperature at lower wall
*T*_*o*_(*K*): Temperature at upper wall
*U*_1_, *U*_2_, *U*_3_(*m*/*s*): Velocity Components
*U*_*v*_(*m*/*s*): Variable Velocity

**Subscripts**
cnt: Carbon Nanotube
*MW*_*cnt*_: Multiple wall carbon nanotube
nf: Nanofluid
*SW*_*cnt*_: Single wall carbon nanotube
