# Supplementary material for: Channelized water driven flow of MHD carbon-nanotube nanofluid influenced by rotation, heat source and thermal radiation
Source: PLoS One. 2023 Dec 27;18(12):e0295406. doi: 10.1371/journal.pone.0295406 (PMC10752567; doi:10.1371/journal.pone.0295406)
Supplement: S1 File — (PDF) [file pone.0295406.s001.pdf]

```
function nanofluid
%f(0) f'(0) f''(0) f'''(0) g(0) g'(0) t(0) t'(0);
%y(1) y(2)   y(3)   y(4)  y(5)  y(6)  y(7) y(8);
clc
clear all
close all
s=[-3 -1 -0.5 0 0.5 1 1.5 5];
a1= [0.2 0.5 0 1 2 3 4 5];
a2= [0.2 0.5 0 1 2 3 4 5];
Phi=[0 0.1 0.25 ];
KCNT=[6600 3000];
Rho=[2600 1600];
CP=[425 796];
phi = Phi(1);
kf = 0.613;
rhof = 997;
Cpf =4179;
kCNT = KCNT(1);
rhoCNT=Rho(1);
cpCNT= CP(1);

S =1;
A1 = 1/2;
A2 = 1;%%%

e1=(1-phi+phi*(rhoCNT/rhof))*(1-phi)^2.5;
e2=1-phi+(phi*(rhoCNT*cpCNT)/(rhof*Cpf));
e3=(1-phi+2*phi*(kCNT/(kCNT-kf))*log((kCNT+kf)/(2*kf)))/(1-phi+2*phi*(kf/(kCNT-kf))*log↙
((kCNT+kf)/(2*kf)));

M = 0;
Nr= 0;
Q = 0;
Bi= 10;
Pr =6.2;

init     = bvpinit(linspace(0,1,100), [0 1 0 0 0 0 1 0]);
sol      = bvp4c(@bvpex11,@bc3D1,init);
x        = sol.x;
y        = sol.y;
%skin     = vpa((y(3,end)*(1-phi+phi*(rhoCNT/rhof))/(1-phi)^2.5),6)
%Nu       = vpa(-y(8,1)*(1+Nr/e3),6)

solution = vpa(-deval(sol,1),6);
figure(1)
plot(x, y(2,:),'--b', 'LineWidth',1)


xlabel('\eta');
ylabel('f(\eta)');
title('A_1=A_2=2, S=1, M=20, Nr=0.5, Q=0.7')
```

```
 hold on
function residual = bc3D1(y1,yinf)

          residual = [y1(1); y1(2)-1; y1(5);y1(7)-1; yinf(1)-S; yinf(2);yinf(5);yinf
(7)];%yinf(8)+Bi*(yinf(7)-1)/e3];
end

    function ysol = bvpex11(x,y)


          yy1    = A1*e1*(y(2)*y(3)-y(1)*y(4))+2*A2*e1*y(6)+M*y(3);

           yy2    = A1*e1*(y(5)*y(2)-y(1)*y(6))-2*A2*e1*y(2)+M*y(5);

           yy3    =(1/(1+(Nr/e3)))*(-Pr*e2*(A1/e3)*y(1)*y(8)-Q*y(7)/e3) ;
           ysol = [y(2); y(3);y(4); yy1; y(6);yy2; y(8); yy3];
    end

end
```
