# Supplementary material for: Channelized water driven flow of MHD carbon-nanotube nanofluid influenced by rotation, heat source and thermal radiation
Source: PLoS One. 2023 Dec 27;18(12):e0295406. doi: 10.1371/journal.pone.0295406 (PMC10752567; doi:10.1371/journal.pone.0295406)
Supplement: S1 Appendix — (PDF) [file pone.0295406.s002.pdf]

Appendix

The governing momentum equations for the given MHD flow are:

$$\frac{\partial \mathbb{U}_1}{\partial x} + \frac{\partial \mathbb{U}_2}{\partial y} = 0, \tag{A.1}$$

$$\rho_{nf}(\mathbb{U}_1 \frac{\partial \mathbb{U}_1}{\partial x} + \mathbb{U}_2 \frac{\partial \mathbb{U}_1}{\partial y} + 2\Omega \mathbb{U}_3) = -\frac{\partial p^*}{\partial x} + \mu_{nf} \frac{\partial^2 \mathbb{U}_1}{\partial y^2} - \sigma_{nf} B_0^2 \mathbb{U}_1, \tag{A.2}$$

$$\rho_{nf}(\mathbb{U}_1 \frac{\partial \mathbb{U}_2}{\partial x} + U_2 \frac{\partial \mathbb{U}_2}{\partial y}) = -\frac{\partial p^*}{\partial y} + \mu_{nf} \frac{\partial^2 \mathbb{U}_2}{\partial y^2}, \tag{A.3}$$

$$\rho_{nf}(\mathbb{U}_1 \frac{\partial \mathbb{U}_3}{\partial x} + U_2 \frac{\partial \mathbb{U}_3}{\partial y} - 2\Omega \mathbb{U}_1) = \mu_{nf} \frac{\partial^2 \mathbb{U}_3}{\partial y^2} - \sigma_{nf} B_0^2 \mathbb{U}_3. \tag{A.4}$$

The following similarity transformations are used to obtain non-dimensional system of equations

$$\mathbb{U}_1 = mxf'(\eta), \;\; \mathbb{U}_2 = -mhf(\eta), \;\; \mathbb{U}_3 = mxg(\eta), \;\; \theta(\eta) = \frac{T - T_0}{T_a - T_0}, \;\; \eta = \frac{y}{h}. \tag{A.5}$$

Various derivatives of U_1, U_2 and U_3 are

$$\frac{\partial \mathbb{U}_1}{\partial x} = mf'(\eta), \quad \frac{\partial \mathbb{U}_2}{\partial y} = -mf'(\eta), \quad \frac{\partial \mathbb{U}_2}{\partial x} = 0, \quad \frac{\partial^3 \mathbb{U}_1}{\partial y^3} = \frac{mxf^{iv}}{h^3}, \tag{A.6}$$

$$\frac{\partial \mathbb{U}_1}{\partial y} = \frac{mxf''(\eta)}{h}, \quad \frac{\partial^2 \mathbb{U}_2}{\partial y^2} = \frac{-mf''(\eta)}{h}, \quad \frac{\partial^2 \mathbb{U}_1}{\partial y^2} = \frac{mxf'''(\eta)}{h^2}, \tag{A.7}$$

To eliminate the partial derivative of p^*, we take the y derivative of Eq. (A.1) and x derivative of Eq. (A.2).

$$\begin{aligned}\rho_{nf}(\mathbb{U}_1 \frac{\partial^2 \mathbb{U}_1}{\partial y \partial x} + \frac{\partial \mathbb{U}_1}{\partial y}\frac{\partial \mathbb{U}_1}{\partial x} + \mathbb{U}_2 \frac{\partial^2 \mathbb{U}_1}{\partial y^2} + \frac{\partial \mathbb{U}_2}{\partial y}\frac{\partial \mathbb{U}_1}{\partial y} + 2\Omega \frac{\partial \mathbb{U}_3}{\partial y}) = -\frac{\partial^2 p^*}{\partial y \partial x} + \mu_{nf} \frac{\partial^3 \mathbb{U}_1}{\partial y^3} \\ - \sigma B_0^2 \frac{\partial \mathbb{U}_1}{\partial y},\end{aligned} \tag{A.8}$$

$$\rho_{nf}(\frac{\partial \mathbb{U}_1}{\partial x}\frac{\partial \mathbb{U}_2}{\partial x} + \mathbb{U}_1 \frac{\partial^2 \mathbb{U}_2}{\partial x \partial y} + \frac{\partial \mathbb{U}_2}{\partial x}\frac{\partial \mathbb{U}_2}{\partial y}) = -\frac{\partial^2 p^*}{\partial x \partial y} + \mu_{nf} \frac{\partial^3 \mathbb{U}_2}{\partial x \partial y^2}, \tag{A.9}$$

By subtracting Eq. (A.8) and Eq. (A.9) and then substituting Eq. (A.5-A.6), we get

$$((1-\phi)\rho + \phi\rho_{Cnt})(\frac{m^2xf'f''}{h} - \frac{m^2xff'''}{h} + \frac{2\Omega mxg'}{h}] = \frac{\mu}{(1-\phi)^{2.5}}(\frac{mxf^{iv}}{h^3}) - \sigma B_0^2 mxf'',$$

$$\begin{aligned}((1-\phi) + \frac{\phi\rho_{Cnt}}{\rho})(1-\phi)^{2.5}[\frac{m^2xf'f''}{h} - \frac{m^2xff'''}{h} + \frac{2\Omega mxg'}{h}] = \frac{\mu}{\rho}(\frac{mxf^{iv}}{h^3}), \\ -\frac{\sigma}{\rho} B_0^2 mxf''(1-\phi)^{2.5},\end{aligned}$$

$$\varepsilon_1[m^2xf'f'' - m^2xff''' + 2\Omega mxg'] = \nu(\frac{mxf^{iv}}{h^2}) - \frac{\sigma}{\rho} B_0^2 mxf''(1-\phi)^{2.5},$$

$$(\frac{h^2}{\nu})\varepsilon_1[m^2xf'f'' - m^2xff''' + 2\Omega mxg'] = mxf^{iv} - \frac{\sigma h^2}{\rho\nu} B_0^2 mxf''(1-\phi)^{2.5},$$

$$f^{iv} - \varepsilon_1\zeta A_1(f'f'' - ff''') - 2A_2\varepsilon_1\zeta g' - \zeta M f'' = 0. \tag{A.10}$$

where,

$$M = \frac{\sigma h^2 B_0^2}{\rho\nu}, \quad \zeta = (1-\phi)^{2.5}, \quad A_1 = \frac{mh^2}{\nu}, \quad A_2 = \frac{\Omega h^2}{\nu}.$$
